# Vaccine confidence in China after the Changsheng vaccine incident: a cross-sectional study

**DOI:** 10.1186/s12889-019-7945-0

**Published:** 2019-11-27

**Authors:** Baohua Liu, Ruohui Chen, Miaomiao Zhao, Xin Zhang, Jiahui Wang, Lijun Gao, Jiao Xu, Qunhong Wu, Ning Ning

**Affiliations:** 1 Department of Social Medicine, School of Health Managment, Harbin Medical University, Harbin, Heilongjiang China; 2 Harbin Center for disease control and prevention, Harbin, Heilongjiang China; 3 Department of Health Management, School of Public Health, Nantong University, Nantong, Jiangsu China

**Keywords:** Vaccine, Confidence, Negative vaccine incident, Vaccine confidence

## Abstract

**Background:**

China’s achievements in immunization are being threatened by a vaccine crisis. This paper aims to investigate vaccine confidence in China after the Changsheng vaccine incident and attempts to identify the factors contributing to it.

**Methods:**

An online cross-sectional investigation was conducted from 1 to 25 September 2018. Descriptive analysis and logistic regression were performed to examine the associations between socio-demographic factors, cognition and attitudes towards the Changsheng vaccine incident and vaccine confidence.

**Results:**

We included 1115 respondents in the final analysis, and found that approximately 70% (783) of the respondents did not have vaccine confidence. More than half of the respondents (54.53%) were dissatisfied with the government’s response measures to the Changsheng vaccine incident. The logistic regression model indicated that vaccine confidence was positively associated with the degree of satisfaction with the government’s response measures (OR = 1.621, 95% CI = 1.215–2.163), attitudes towards the risks and benefits of vaccination (OR = 1.501, 95% CI = 1.119–2.013), concerns about vaccine safety (OR = 0.480, 95% CI = 0.317–0.726), and vaccine efficacy (OR = 0.594, 95% CI = 0.394–0.895).

**Conclusions:**

A majority of the respondents held negative attitudes towards vaccines after the Changsheng vaccine incident. A coordinated effort is required to restore public confidence in vaccines, especially in China, where a nationwide mandatory immunization policy is implemented. To end dissent towards inoculation, a series of actions is crucial and multiple parties should work together to advance efforts and explore the possibility of establishing an open and transparent regulatory system.

## Background

Inoculation has been valued as one of the most effective public intervention measures for safeguarding health [1–3]. However, the success of the cumulative health effects of vaccines also arouses higher vaccine concerns [4–6]. Although efforts have been made to improve the safety and purity of vaccines and reduce the number of required injections via combined vaccines [3], public confidence in vaccines and vaccination coverage are still exhibiting declining tendencies in several countries. This has led to the recrudescence and the outbreak of many vaccine-preventable diseases worldwide [4, 7–11].

The lack of confidence in vaccination is not a new phenomenon. The fear and trepidation of vaccination are as old as the vaccine itself [12–15]. There have been a series of vaccine scandals and scares all over the world in the past few decades, which have eroded public confidence and led to devastating results [1]. In 1955, about 220,000 children in the United States were vaccinated against polio. Approximately 200 of these children suffered from paralytic poliomyelitis, and 10 died as a result of inadequate poliovirus inactivation [16]. This is one of the gravest vaccine incidents in American medical history, which—along with other negative events—led to stricter standards and controls for vaccines and prompted the establishment of a vaccine adverse reaction monitoring system in the United States.

Inoculation-related incidents do not even have to be real to have a genuine effect on public opinion. In 1998, a paper published in the *Lancet* suggested that the measles, mumps, and rubella (MMR) vaccine had an association with autism [17]. Even though the causal association between the MMR vaccine and autism was ultimately proven to be non-existent by other studies [18, 19], the widespread dissemination of the original research had profound negative effects on vaccination. Public confidence in immunization is highly variable, and driven by a multitude of determinants such as religion, politics, economics, history, health beliefs, and genuine safety issues [1], among others, vaccine-related negative incidents can be considered a direct and powerful factor that affect vaccine confidence.

Remarkable achievements have been made in the field of immunization since the founding of the People’s Republic of China. A national planned immunization policy for children was started in 1978 [20]. Smallpox and polio have been eradicated nationwide, and the last case of diphtheria was reported in 2006 [21]. In 1983, the inoculation coverage rates of the recommended Bacillus Calmette-Guerin (BCG) vaccine, Diphtheria, Pertussis, and Tetanus (DPT) vaccine, Oral Polio vaccine (OPV) vaccine, and Measles vaccine were 34, 58, 79, and 78%, respectively, while the coverage rate of these vaccines all exceeded 95% nationwide by 2013. Additionally, the number of free vaccines in the immunization program has increased from four to eleven [21]. However, these achievements have been shaken by one major negative vaccine incident in China. In November 2017, the titer indicators of the sample-test DPT vaccines produced by Changchun Changsheng Biotechnology and Wuhan Institute of Biological Products were detected to be substandard based on the requirements of the National Institute of Food and Drug Control. The Changsheng Biotechnology Company produced 252,600 doses of these unqualified DPT vaccines, while the Wuhan Company produced 400,250 doses; a portion of these vaccines was injected. Less than one year later, Changsheng Biotechnology was once again reported for severe violations: this time, the issue involved the production of freeze-dried human rabies vaccine. The company’s falsification of records and unauthorized modification of production processes violated the ‘Pharmaceutical Production Quality Management Standard’.

Negative information about vaccine is uniquely attractive to the media. The news quickly spread across the Internet and social media and aroused fear and panic regarding the possible side effects of such faulty vaccines, which ultimately led to a heated nationwide discussion and widespread international attention. According to Sina Weihotspot (http://www.wrd.cn/login.shtml), from 8 July 2018 to 5 August 2018, more than 8057 pieces of vaccine-related information were disseminated online. Most of the information (61%) was sensitive and emotional, and 1.08% came from abroad. The amount of information peaked on July 23, 2018 when a total of 2445 pieces of text information were generated, and the sound volume (measured by the amount of message forwarding, thumbs-ups and comments) was as high as 1,526,664.

The Changsheng vaccine incident in China directly escalated into a health trust crisis, which triggered a climate of nationwide public dissent. The vaccines involved in the incident were evaluated by a panel of experts from the State Council of China, which concluded that the injection of these impotent vaccines had neither a protective effect nor side effects. Although there were no records indicating any negative reactions or mortality caused by substandard DPT or rabies vaccines, they were reported to be toxic by some social media [22], and there were rumours that the faulty rabies vaccines caused actual dog-bite deaths.

In addition to widespread fear and indignation, the vaccine crisis led to a universal questioning of vaccines and severely threatened the achievements in immunization in China that were accumulated over the past decades [21]. To reverse this situation, measures should be taken to restore and sustain public confidence of vaccines in China, moreover, understanding the factors related to vaccine confidence will be a touchstone for a more standardized vaccine regulatory system and a sustainable immunization program in China. The aim of this study was to investigate vaccine confidence among Chinese people after such a vaccine crisis, as well as to identify the contributing factors associated with it before they evolve into a sustained decline in immunization coverage. The present study will also provide evidence for the establishment of a high-standard vaccine regulatory system in China in the future.

## Methods

### Questionnaire design

We developed a self-administered questionnaire by conducting sufficient literature research work and obtaining expert-advice. In addition to specific questions based on the Changsheng vaccine incident, our study also incorporated many ideas from previous research [2, 14, 15, 23–27]. To ensure the surface and content validity of the questionnaire, we invited seven experts from the fields of immunization, drug regulation, health administration, epidemiology, and crisis management to review the questionnaire three times. Additionally, we selected 50 people to form a group doing a two-week test-retest reliability test (Spearman correlation r = 0.79). The questionnaire contained the following sections: (1) Socio-demographic information; (2) Cognition and attitudes towards vaccines and the vaccine incident; (3) Incident response assessment.

The dependent variable of this study was the respondents’ self-assessed confidence in vaccines, which was evaluated by the item: ‘Do you have confidence in vaccines in China?’ using a five-point Likert scale ranging from 1 (completely confident) to 5 (not confident at all). For the purposes of modelling, we created a dichotomous variable in the hope of comparing respondents who had a positive attitude towards vaccines with those who did not. The respondents who scored 1 and 2 on the Likert scale were classified as ‘confident’, and those with scores 3, 4, 5 were classified as ‘not confident’.

Socio-demographic data were collected for participants’ gender, age, educational level, residential area, marital status, monthly family income, and whether they had one or more children under seven years old.

Participants were asked to assess issues related to vaccines and the Changsheng vaccine incident, including ‘The consequences of the failure of the DTP vaccination/rabies vaccination’, and using questionnaire items such as ‘Are you concerned about vaccine efficacy/safety’ and ‘Is there anyone around you who strongly opposes vaccination?’. Respondents’ awareness regarding the role and risks of vaccines was ascertained by four items, including ‘Vaccination risks outweigh benefits’, ‘Vaccines are the most effective way of fighting infectious diseases’, ‘Collapse of immunization defence can lead to outbreaks of infection diseases’, and ‘Identifying the root of an incident and strengthening regulations are more important than punishments’.

The item ‘Satisfied with the response to this vaccine incident’ was specifically developed for this study. The degree of satisfaction with the crisis response was assessed by ten items on the investigation of cases, including: national leaders’ attention, transparency of case information, accountability to the drug regulatory authorities, accountability to the government and the regulatory authority officials involved, punishment of enterprises, the legal responsibility of enterprises, re-vaccination and follow-up observation for problematic vaccinations, risk communication, and compensation. These items were designed based on literature research and developed by an expert group. Each item was given a score from 1 to 5, from extremely unsatisfied to extremely satisfied, with a total score ranging from 10 to 50. Respondents were classified into two groups based on the median score: ≤30, unsatisfied; and > 30, satisfied.

### Sample and data collection

The cross-sectional survey was conducted via an online investigation website- Wenjuanwang (www.wenjuan.com), which is widely used in China. The questionnaire was transposed to online access via the survey site, and then the platform generated a link for respondents to access. In order to increase the representation of geographical distribution, we selected three provinces each in the eastern (Shandong, Zhejiang, Hebei), central (Heilongjiang, Anhui, Hunan), and western (Sichuan, Chongqing, Yunan) regions of China.

Based on the Gross Domestic Product (GDP) ranking of cities in their province, the cities in each province were divided into two groups: economically developed and not well developed. ‘Economically developed’ refers to cities with higher economic development levels than the average GDP level of all cities in the province; those with GDP levels lower than the average were classified into the ‘not well developed’ group. We selected one developed city and one not well-developed city in each province. In line with the willingness to cooperate among community doctors and village doctors, one community health service center in an urban area and one village clinic in a rural area within each sample city were selected (see Additional file 1). The questionnaire links were sent by the doctors to their community’s WeChat groups. People in the group could voluntarily decide whether or not to respond. WeChat (Weixin in China) is the most popular social network in China [28, 29]. According to Tencent (WeChat Data Reporting) [30], there were more than one billion active WeChat users as of September 2018.

Data were collected from September 1 to 25, 2018. The identifiers of the respondents were not collected to ensure confidentiality throughout the study. Only completed questionnaires could be uploaded to the survey platform. According to the background observation of the website, 1926 people clicked on the link, and a total of 1203 people filled out the questionnaire. The research team reviewed the questionnaires carefully and excluded participants under the age of 18 and those with the same answers for every question. A total of 1115 valid questionnaires were finally collected (effective response rate = 92.68%). Respondents were almost evenly distributed across eastern (392), central (363), and western (360) China.

### Data analysis

A descriptive analysis was adopted to show respondents’ demographic characteristics, cognition, and attitudes towards the Changsheng vaccine incident and vaccines. Chi-square tests were performed to assess whether the independent variables were statistically significant and a logistic regression was determined to filter the factors affecting vaccine confidence (performed using SPSS 22.0). The significance level was set at 0.05.

## Results

### Characteristics of the respondents

More than half (59.28%) of the respondents were women. The ages of the respondents ranged from 18 to 74 years, with an average age of 33.6. Approximately 61.08% of respondents were married, and 59.46% of respondents have received college degree or higher. Approximately 29.78% (*n* = 332) of respondents reported positive confidence in vaccines (Table 1).

**Table 1:** Socio-demographic characteristics of respondents (*n* = 1115)

Characteristics	Total	Confidence in vaccines		χ^2^	*P*-value
	N (%)	Confident (*n* = 332)	Not confident (*n* = 783)		
Gender				1.190	0.275
Male	454(40.72)	127(38.25)	327(41.76)		
Female	661(59.28)	205(61.75)	456(58.24)		
Age				7.485	0.112
≤20	36(3.22)	12(3.61)	24(3.07)		
21–30	520(46.64)	172(51.81)	348(44.44)		
31–40	283(25.38)	68(20.48)	215(27.46)		
41–50	189(16.95)	55(16.57)	134(17.11)		
≥51	87(7.81)	25(7.53)	62(7.92)		
Education background				5.872	0.118
High school graduate or lower	275(24.66)	85(25.60)	190(24.27)		
Junior college	177(15.87)	43(12.95)	134(17.11)		
College graduate	440(39.46)	126(37.95)	314(40.10)		
Master degree and above	223(20.00)	78(23.49)	145(18.52)		
Living area				0.036	0.849
Urban area	805(72.20)	241(72.59)	564(72.03)		
Rural area	310(27.80)	91(27.41)	219(27.97)		
Family income per month				6.883	0.142
≤7000 Yuan	122(10.94)	40(12.05)	82(10.47)		
7001–10,000 Yuan	248(22.24)	67(20.18)	181(23.12)		
10,001–13,000 Yuan	286(25.65)	76(22.89)	210(26.82)		
13,001–16,000 Yuan	227(20.36)	66(19.88)	161(20.56)		
> 16,000 Yuan	232(20.81)	83(25.00)	149(19.03)		
Marital status				3.011	0.222
Single	392(35.16)	127(38.25)	265(33.84)		
Married	681(61.08)	196(59.04)	485(61.94)		
Separated/divorced/widowed	42(3.76)	9(2.71)	33(4.22)		
Have one or more children under 7 years of age				0.281	0.596
Yes	362(32.47)	104(31.33)	258(32.95)		
No	753(67.53)	228(68.67)	525(67.05)		

### Cognition and attitudes towards vaccines and the Changsheng vaccine incident

Overall, 24.04% of respondents indicated that someone around them strongly opposed vaccination. About four-fifths of respondents (79.01%) assumed that the consequences of the substandard DPT vaccination were serious, and more respondents (88.07%) considered the inefficacy of the rabies vaccination to be a serious issue. Over three-fifths of respondents expressed their worry about vaccine efficacy (66.37%) and safety (65.20%). In addition, over half of the respondents (54.53%) were not satisfied with the government’s response to the incident. Most respondents believed vaccination to be the best means of fighting infectious diseases (61.08%), and they thought that the collapse of immunization defence could lead to infectious diseases outbreaks (68.58%). About 68.97% emphasised identifying the root of the incident and strengthening regulations rather than punishments. Overall, 52.38% disagreed that the benefits of vaccines outweighed the risks (Table 2).

**Table 2:** Cognition and evaluation of the Changsheng vaccine incident

	Total	Confidence in vaccines		X^2^	*P*-value
	N (%)	Confident(*n* = 332)	Not confident(*n* = 783)		
Someone around you strongly opposes vaccination				0.114	0.736
Yes	268(24.04)	82(24.70)	186(23.75)		
No	847(75.96)	250(75.30)	597(76.25)		
The consequences of the failure of the DTP Vaccination				4.592	0.032^*^
Serious	881(79.01)	249(75.00)	632(56.72)		
Not serious	234(20.99)	83(25.00)	151(19.28)		
The consequences of the failure of rabies vaccination				8.464	0.004^**^
Serious	982(88.07)	278(83.73)	704(89.9)		
Not serious	133(11.93)	54(16.27)	79(10.09)		
Satisfied with the response to this vaccine event				30.568	0.000^**^
Satisfied	507(45.47)	193(58.13)	314(40.10)		
Not satisfied	608(54.53)	139(41.87)	469(59.90)		
Concern about vaccine efficacy				65.404	0.000^**^
Worried	740(66.37)	162(49.80)	578(73.82)		
Not worried	375(33.63)	170(51.20)	205(26.18)		
Concern about vaccine safety				71.431	0.000^**^
Worried	727(65.20)	155(46.69)	572(73.05)		
Not worried	388(34.80)	177(53.31)	211(26.95)		
Vaccine is the most effective way of fighting infectious diseases				9.734	0.002^**^
Agree	681(61.08)	226(68.07)	455(58.10)		
Disagree	434(38.92)	106(31.93)	328(41.89)		
The collapse of immunization defence can lead to outbreaks of infection diseases				5.237	0.024^*^
Agree	765(68.61)	244(73.49)	521(66.54)		
Disagree	350(31.39)	88(26.51)	262(33.46)		
Identifying the root of incident and strengthening regulations are more important than punishments				9.721	0.002^**^
Agree	769(68.97)	251(75.61)	518(66.16)		
Disagree	346(31.03)	81(24.39)	265(33.84)		
Vaccination benefits outweigh risks				6.136	0.013^*^
Agree	531(47.62)	177(53.33)	354(45.21)		
Disagree	584(52.38)	155(46.67)	429(54.79)		

^**^P < 0.01; ^*^P < 0.05

### Responses to the Changsheng vaccine incident

**Fig. 1:**
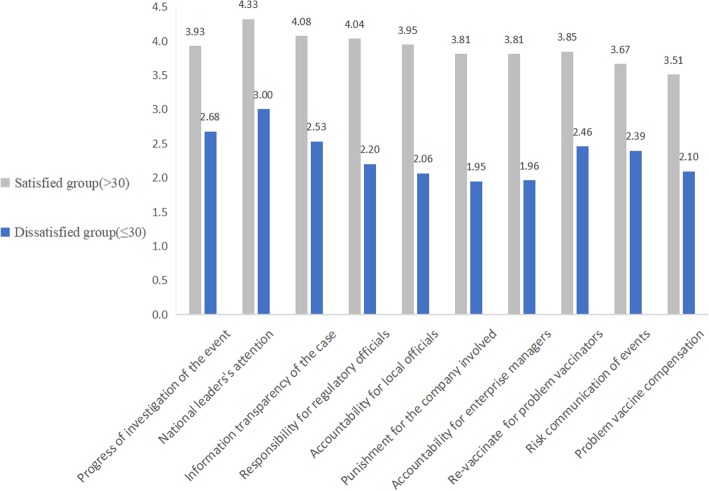
Satisfaction with the measures taken to deal with the Changsheng vaccine incident. The satisfaction with crisis response was assessed by 10 items. Each item was given a score from 1 to 5, from extremely unsatisfied to extremely satisfied, with a total score range from 10 to 50; respondents were classified into two groups based on the median score: ≤30, unsatisfied; > 30, satisfied

To better understand the respondents’ attitudes to the handling measures of the Changsheng vaccine incident, we classified them into two groups: the satisfied (> 30) and unsatisfied (≤30) group (Fig. 1). This distinction was made based on whether the sum of 10 response items fell above or below the mean value. The satisfied group tended to be most content with the top national leaders’ attention (4.33), followed by the progress of the event investigation (4.08), and responsibility of the regulatory officials (4.04). The dissatisfied group expressed general dissatisfaction with the series of punishments for vaccine fraud, for instance, the punishment for the company involved (1.95), accountability of enterprise managers (1.96), and accountability of local officials (2.06). Like the satisfied group, the most satisfactory response was for top national leaders’ attention (3.00).

### Multivariable analysis

Table 3 displays the results of the analysis of the dominant variables in the binary logistic regression model. The respondents who were more satisfied with the response to the incident (OR = 1.621, 95% CI = 1.215–2.163) and those who agreed that the benefits of vaccination outweighed the risks (OR = 1.501, 95% CI = 1.119–2.013) tended to hold confidence in vaccines. The respondents who expressed concern about the efficacy (OR = 0.594, 95% CI = 0.394–0.895) and safety (OR = 0.480, 95% CI = 0.317–0.726) were inclined to lose confidence in vaccines.

**Table 3:** Multivariable logistic regression analysis of factors affecting vaccine confidence

	β	S.E	Wald	P	Odds Ratio (95% CI)
Gender	0.254	0.146	3.035	0.081	1.289 (0.969–1.714)
Living area	−0.009	0.164	0.003	0.956	0.991(0.719–1.366)
Age					
≤20					1.000
21–30	−0.004	0.396	0.000	0.992	0.996 (0.458–2.164)
31–40	−0.262	0.411	0.408	0.523	0.769 (0.344–1.721)
41–50	−0.215	0.421	0.261	0.610	0.807 (0.354–1.840)
≥51	−0.287	0.466	0.380	0.538	0.750 (0.301–1.871)
Education level					
High school graduate or lower					1.000
Junior college	−0.243	0.239	1.035	0.309	0.784 (0.490–1.253)
College graduate	−0.196	0.197	0.997	0.318	0.822 (0.559–1.208)
Master degree and above	−0.092	0.240	0.148	0.701	0.912 (0.570–1.459)
Family income per month					
≤7000 Yuan					1.000
7001–10,000 Yuan	−0.222	0.259	0.737	0.391	0.801 (0.482–1.330)
10,001–13,000 Yuan	−0.255	0.257	0.981	0.322	0.775 (0.468–1.283)
13,001–16,000 Yuan	−0.035	0.267	0.017	0.896	0.966 (0.573–1.629)
> 16,000 Yuan	0.181	0.263	0.475	0.491	1.199 (0.716–2.008)
The consequences of the failure of the DTP vaccine	0.115	0.200	0.327	0.567	1.121 (0.757–1.661)
The consequences of the failure of rabies vaccine	−0.405	0.248	2.663	0.103	0.667 (0.410–1.085)
Satisfied with the response to this vaccine event	0.483	0.147	10.792	0.001^**^	1.621 (1.215–2.163)
Concern about vaccine efficacy	−0.521	0.210	6.191	0.013^*^	0.594 (0.394–0.895)
Concern about vaccine safety	−0.734	0.211	12.074	0.001^**^	0.480 (0.317–0.726)
Vaccines are the most effective means of fighting infectious diseases	0.211	0.168	1.584	0.208	1.235 (0.889–1.716)
The collapse of immunization defence can lead to outbreaks of infection diseases	0.177	0.186	0.908	0.341	1.194 (0.829–1.720)
Identifying the root of an incident and strengthening regulations are more important than punishments	0.227	0.186	1.496	0.221	1.255 (0.872–1.805)
Vaccination benefits outweigh risks	0.406	0.150	7.340	0.007^**^	1.501 (1.119–2.013)

95% CI = 95% confidence interval; ^*^0.01 < *p*<0.05; ^**^P < 0.01

## Discussion

Decades of painstaking efforts have made China’s planned immunization one of the largest in the world. Smallpox, polio and diphtheria have been eliminated nationwide, and the incidence of other vaccine-preventable infectious diseases is comparable to the levels in developed countries [21]. However, in this study, only 29.78% of respondents reported positive confidence in vaccines after the crisis occurred.

We found that vaccine confidence was significantly associated with participants’ concerns regarding vaccine safety and efficacy, satisfaction with incident response, and perception of vaccine benefits versus vaccine risks. Similar to previous studies [31], we also found that cognitive factors played a more significant role than socio-demographic ones in the social differentiation of vaccine confidence. In this study, no significant associations were found between socio-demographic factors and vaccine confidence. A systematic review on vaccination attitudes in different countries showed that the relationships between vaccine attitude and socio-demographic factors, such as income and education, were varied and were not explained well enough [27].

To better understand the underlying relationships, we further explored whether perceptions and cognition of vaccines varied at different levels of income and education (see Additional file 2: Table S1-S9). We found that, to some extent, the levels of income and education did affect respondents’ cognition and recognition of vaccines. For example, compared to respondents with the lowest income and the lowest education levels, people with higher levels of income and education were more in agreement with the view that the ‘The collapse of immunization defence can lead to outbreaks of infection diseases. However, among the four influencing factors related to vaccine confidence that were identified in the multivariate analysis, only ‘Vaccination benefits outweigh risks’ was significantly associated with income and education, furthermore, the relationships between income and education and the other three factors were either non-significant or not completely clear.

### The concern about vaccine efficacy and safety

Vaccination is expected to be a safety health intervention that can effectively prevent target infectious diseases. Vaccine-related adverse incidents will therefore undoubtedly arouse and aggravate concerns about the quality of vaccines and their regulation [5, 32]. In our study, we found that people who expressed concerns about vaccine efficacy or safety were more likely to lose confidence in vaccines, which matches the trends observed in past research [33]. In Stephen Black’s study [34], he expressed that a successful establishment of vaccine confidence cannot depend only on an appropriate vaccine delivery system, but that it should be combined with vaccine efficacy and safety; he indicated that this is the only way to enhance the vaccine acceptance and utilization.

Parental concern about the efficacy of vaccines could result in negative attitudes, because there is no guarantee that vaccines can absolutely prevent the vaccine-preventable diseases [35]. As a result of evidence like that, an unstoppable vaccine-confidence decline occurred [36]. Vaccine inefficacy was the key problem in the Changsheng vaccine incident, which was reported as a concern by a majority of the respondents in our study.

Safety has always been the most concerning area within vaccination [14]. However, the achievements and contributions attributed to vaccines resulted in the creation of a proverb— ‘Vaccines are victims of their own success’ [15, 37]. This is evident by the declining awareness and concern about the seriousness of vaccine-preventable diseases as well as the increased emphasis on the safety of the vaccines themselves [38], especially under the birth-control policy in China. Moreover, some vaccine-preventable diseases have been eliminated from the lives of young and middle-aged individuals, which—to some extent—may have led to the increased focus on the safety of vaccines. The perception of vaccine safety is probably rooted in cultural backgrounds, such as specific cognitive and religious beliefs [39]. Larson et al. [7] reported that different regions were inclined to perceive vaccine safety in diverse ways. Low vaccination rates may have connections with insufficient infrastructure or resources, and also with a lack of confidence in vaccines. Further, low confidence in vaccines could stem from perceptions regarding vaccine safety [40]. Although the Changsheng vaccine crisis did not involve adverse reactions or other vaccine-safety issues, people still showed a high level of concern.

### Risks and benefits of vaccination

The risks of vaccination are actually safety issues, while its benefits are based on safe and effective vaccination [41]. In our study, many respondents admitted that vaccines do have a positive impact on health. However, a higher percentage of participants still believed that the risks outweighed the benefits. Our study also revealed that respondents who supposed that the benefits outweighed the risks were inclined to hold confidence in vaccines, which was in line with the findings of Betsch and Sachse’s [42]. Additionally, our research showed that 72.6% of the respondents (*n* = 810) deemed health risks from vaccination to be an important issue that hindered the progress of China’s planned immunization of children (data are not shown in any table).

A qualitative study found that health professionals supported vaccination and immunization program, but expressed general anxiety about specific details, including the risks of vaccination [43]. In order to improve people’s confidence in vaccines, extensive health education should be provided to make people aware of the benefits of vaccines; in addition, considerable efforts should be taken to ensure the vaccine safety and quality [44].

### Satisfaction with the response to the Changsheng vaccine incident

In our study, we used several dimensions to measure participants’ satisfaction with the government’s response to this incident. The results showed that people who were highly satisfied with the corresponding responses tend to hold confidence in vaccines. The respondents were most satisfied with the top national leaders’ (Xi Jinping and Li Keqiang) concern and the great importance these leaders attached to this incident. The incident was ordered to be thoroughly investigated and those responsible were held accountable. These actions could convince the anxious and angered public that the incident would be taken seriously. Additionally, these actions allowed people to see that a health-related incident is prioritized highly.

The transparency of information related to the case also played a crucial role in the incident response, owing to the continuous expansion of the network news media and the diversification of information access channels. Related information was updated immediately, which satisfied the public’s thirst for information. Furthermore, the government has an obligation to ensure that information related to the case is transparent and available in a timely manner, thus allowing the general public to monitor the progress and ensure equity and justice in the process of handling this crisis [45].

This study revealed that the most unsatisfactory measures were a series of punishments and accountability measures for the enterprises, business managers, and relevant officials. People were eager to see severe penalties meted out for those who had violated the rules. In our study, 83.3% of respondents assumed that ‘Increasing penalties for those enterprises that conducted illegal production and implementing huge penalties’ would help increase the public’s confidence in vaccines, and 79.7% of respondents thought that ‘Strong supervision of vaccine access, production, and circulation’ would also be beneficial (data are not shown in any table). In addition, to appease the widespread public anger and panic, severe penalties can provide a powerful deterrent to producing disqualified vaccines and can ensure better regulation.

It is the government’s obligation to shoulder the responsibility and prioritize civil rights and health. Re-vaccination, clinical observation, and compensation are effective approaches for maintaining confidence in vaccinations that have had a large public impact [46]. In China, the planned immunization policy is mandatory, and re-vaccination, clinical observation, and compensation will assure the public that if something goes wrong, the government will stand up to protect their civil rights. Further, we found ‘accountability’ to be of great importance to the respondents. There is no accountability to speak of when the laws and regulations are absent; thus, specific legislative and disposal measures will offer a standard to be followed, and will restore public faith in the vaccine program. This will facilitate quicker restoration of the public’s faith in the vaccination programs and will ensure greater accountability in the future.

### Attitudes towards vaccination by nearby people

A previous study [1] has shown that the opinions and attitudes of other people could greatly predict individuals’ confidence in vaccines. However, the variable ‘Having someone around strongly opposed to vaccination’ was not statistically significant in our study, and only 24.04% of the respondents indicated that someone around them strongly opposed vaccination after this incident. The Chinese Planned immunization Policy could partly explain this result. The mandatory requirements of this model can provide some assistance in reducing the indefinite level of variability in personal vaccine attitudes [47].

The factors that affect attitudes towards vaccines are complicated but interrelated; therefore, in order to explore the bigger picture of vaccine confidence, it is essential to include all related elements into a complex linked system. Thus, under no circumstances shall we put the concerns regarding vaccine safety and efficacy into an isolated causal chain because communication with friends, press reports, information from a variety of sources [48], government policies, and even the bigger global picture are all factors that potentially influence public confidence in vaccines. For instance, when the Changsheng vaccine incident exploded, social media and the press began paying a substantial amount of attention to the topic, and opinion leaders and various media outlets freely presented their opinions. The public tends to reap information that perfectly matches their appetite, which somehow will have a potential impact on attitudes towards vaccines [49].

As of 2 February 2019, 48 government officials involved in the Changsheng vaccine incident have been seriously punished, and the company was fined 9.1 billion Yuan. Eighteen company personnel who violated criminal law were arrested, including the company’s chairman. The Vaccine Management Law (Draft), which was discussed and approved at a State Council executive meeting, has recently levied public opinion, and the objective of this new vaccine law is to ensure the safety, efficacy, and accessibility of vaccines.

While we were conducting this cross-sectional survey, the vaccine crisis had just been unveiled and the official investigation on the incident was still underway. Consequently, many of the above-mentioned punishments had not yet been executed. The later progress in early 2019 demonstrated that both the Chinese government and society had tried their best to mend its fences for regaining public confidence in vaccines.

This research has some interesting findings and implications, but there are some limitations that need to be acknowledged. First, the use of a cross-sectional survey may have limited causal inference. In future research, longitudinal studies can be used to further examine the causal relationships. Second, our research was closely associated with the Changsheng vaccine incident, and there may be other factors affecting he public’s confidence in vaccines. Third, the survey was conducted via a social network media, and most respondents were relatively well-educated, had higher incomes, and lived in urban areas. These factors limit the generalizability of the findings, and the results of this study should be interpreted with caution. However, WeChat users were valuable to our study in the context of the Changsheng vaccine incident. This is because information on the Changsheng vaccine incident was mainly disseminated and attracted people’s concerns through networks and social media, and WeChat is the most popular social network in China. ‘Wenjuanxing’ and the use of WeChat allowed the questionnaire to be quickly distributed and collected. In addition, because the data collection was voluntary, we believe the results were more truthful and reliable. Additionally, previous research has shown that social media can fill in many gaps related to vaccine refusal left by traditional research methods [50].

## Conclusion

As expected, a majority of the respondents showed lower confidence in vaccines after the Changsheng vaccine incident. To end the distrust and dissent towards inoculation, a series of actions are crucial and multiple parties should work together to advance efforts that could possibly lead to future changes. The public crisis caused by this vaccine incident has actually accelerated the reform of China’s vaccine management and has especially sped up its related legislature work; however, this progress is certainly insufficient, and all the vaccine regulators at the different government levels, the vaccine producers, and the vaccine sellers are also obliged to make their own changes. A truly open and transparent regulatory system should be established, and punishment for negative vaccine incidents as a part of post-event control should be taken and implemented seriously.

## Supplementary information

**Additional file 1: Table S1.** Participants’characteristics in the sampling sites.

**Additional file 2: Table S1.** The first cognitive factor "The consequences of the failure of the DTP Vaccination". **Table S2.** The second cognitive factor "The consequences of the failure of rabies vaccination". **Table S3.** The third cognitive factor "Satisfied with the response to this vaccine event". **Table S4'** The fourth cognitive factor "Concern about vaccine efficacy". **Table S5.** The fifth cognitive factor "Concern about vaccine safety". **Table S6.** The sixth cognitive factor “Vaccine is the most effective way of fighting infectious diseases”. **Table S7.** The seventh cognitive factor "Collapse of immunization defence can lead to outbreaks of infection diseases". **Table S8.** The eighth cognitive factor "Identifying the root of incident and strengthening regulations are important than punishments". **Table S9.** The ninth cognitive factor "Vaccination benefits outweigh risks".

## Data Availability

The datasets generated and analyzed during the current study are not publicly available due to privacy restrictions. Respondents were informed during the consent process that the data they provide would be available only to the Harbin Medical University.

## Abbreviations

BCG: Bacillus Calmette-Guerin
CI: Confidence Interval
DPT: Diphtheria, Pertussis, and Tetanus
GDP: Gross Domestic Product
MMR: Measles, Mumps, and Rubella
OPV: Oral Polio Vaccine
OR: Odds Ratio
