# Supplementary material for: Vaccine confidence in China after the Changsheng vaccine incident: a cross-sectional study
Source: BMC Public Health. 2019 Nov 27;19:1564. doi: 10.1186/s12889-019-7945-0 (PMC6880575; doi:10.1186/s12889-019-7945-0)
Supplement: Supplementary file 1 — Additional file 1: Table S1. Participants’characteristics in the sampling sites. [file 12889_2019_7945_MOESM1_ESM.doc]

**Table 1** Participants' characteristics in the sampling sites.

| **Characteristics** | **Eastern (392)** | | | | | | | |  | **Central (363)** | | | | | | | |  | **Western (360)** | | | | | | | | **Total** |
| --- | --- | --- | --- | --- | --- | --- | --- | --- | --- | --- | --- | --- | --- | --- | --- | --- | --- | --- | --- | --- | --- | --- | --- | --- | --- | --- | --- |
|  | **Zhejiang** | |  | **Shandong** | |  | **Hebei** | |  | **Anhui** | |  | **Hunnan** | |  | **Heilongjiang** | |  | **Sichuan** | |  | **Yunnan** | |  | **Chongqing** | |  |
|  | **Ningbo** | **Quzhou** |  | **Qingdao** | **Heze** |  | **Shijiazhuang** | **Qinhuangdao** |  | **Wuhu** | **Huangshan** |  | **Zhuzhou** | **Yiyang** |  | **Harbin** | **Hegang** |  | **Chengdu** | **Meishan** |  | **Dali** | **Lijiang** |  | **Shapingba** | **Liangping** |  |
| **Total number** | 68 | 67 |  | 64 | 56 |  | 62 | 75 |  | 58 | 66 |  | 60 | 64 |  | 61 | 54 |  | 55 | 58 |  | 61 | 66 |  | 63 | 57 | 1115 |
| **Gender** |  |  |  |  |  |  |  |  |  |  |  |  |  |  |  |  |  |  |  |  |  |  |  |  |  |  | 0 |
| Male | 26 | 40 |  | 18 | 26 |  | 21 | 33 |  | 22 | 28 |  | 20 | 24 |  | 20 | 23 |  | 29 | 16 |  | 28 | 24 |  | 34 | 22 | 454 |
| Female | 42 | 27 |  | 46 | 30 |  | 41 | 42 |  | 36 | 38 |  | 40 | 40 |  | 41 | 31 |  | 26 | 42 |  | 33 | 42 |  | 29 | 35 | 661 |
| **Age** |  |  |  |  |  |  |  |  |  |  |  |  |  |  |  |  |  |  |  |  |  |  |  |  |  |  | 0 |
| ≤20 | 0 | 1 |  | 4 | 3 |  | 3 | 1 |  | 9 | 4 |  | 0 | 1 |  | 0 | 0 |  | 1 | 4 |  | 1 | 2 |  | 0 | 2 | 36 |
| 21-30 | 35 | 29 |  | 28 | 29 |  | 37 | 44 |  | 25 | 26 |  | 19 | 22 |  | 33 | 18 |  | 22 | 30 |  | 27 | 30 |  | 30 | 36 | 520 |
| 31-40 | 21 | 21 |  | 9 | 8 |  | 10 | 10 |  | 15 | 24 |  | 22 | 21 |  | 20 | 17 |  | 23 | 10 |  | 14 | 13 |  | 16 | 9 | 283 |
| 41-50 | 7 | 13 |  | 10 | 13 |  | 9 | 14 |  | 7 | 9 |  | 14 | 13 |  | 4 | 11 |  | 7 | 9 |  | 12 | 18 |  | 11 | 8 | 189 |
| ≥51 | 5 | 3 |  | 13 | 3 |  | 3 | 6 |  | 2 | 3 |  | 5 | 7 |  | 4 | 8 |  | 2 | 5 |  | 7 | 3 |  | 6 | 2 | 87 |
| **Education background** |  |  |  |  |  |  |  |  |  |  |  |  |  |  |  |  |  |  |  |  |  |  |  |  |  |  | 0 |
| < Bachelor degree | 20 | 16 |  | 28 | 27 |  | 26 | 35 |  | 23 | 28 |  | 24 | 36 |  | 17 | 29 |  | 21 | 32 |  | 24 | 24 |  | 28 | 14 | 452 |
| ≥Bachelor degree & higher | 48 | 51 |  | 36 | 29 |  | 36 | 40 |  | 35 | 38 |  | 36 | 28 |  | 44 | 25 |  | 34 | 26 |  | 37 | 42 |  | 35 | 43 | 663 |
| **Living area** |  |  |  |  |  |  |  |  |  |  |  |  |  |  |  |  |  |  |  |  |  |  |  |  |  |  | 0 |
| Urban area | 57 | 52 |  | 52 | 31 |  | 45 | 50 |  | 44 | 53 |  | 46 | 46 |  | 54 | 33 |  | 38 | 30 |  | 43 | 50 |  | 44 | 37 | 805 |
| Rural area | 11 | 15 |  | 12 | 25 |  | 17 | 25 |  | 14 | 13 |  | 14 | 18 |  | 7 | 21 |  | 17 | 28 |  | 18 | 16 |  | 19 | 20 | 310 |
| **Family income per month** |  |  |  |  |  |  |  |  |  |  |  |  |  |  |  |  |  |  |  |  |  |  |  |  |  |  | 0 |
| ≤10000 yuan | 11 | 13 |  | 23 | 19 |  | 30 | 37 |  | 10 | 11 |  | 24 | 20 |  | 31 | 29 |  | 10 | 23 |  | 24 | 19 |  | 16 | 20 | 370 |
| > 10000 yuan | 57 | 54 |  | 41 | 37 |  | 32 | 38 |  | 48 | 55 |  | 36 | 44 |  | 30 | 25 |  | 45 | 35 |  | 37 | 47 |  | 47 | 37 | 745 |
| **Marital status** |  |  |  |  |  |  |  |  |  |  |  |  |  |  |  |  |  |  |  |  |  |  |  |  |  |  | 0 |
| Single | 20 | 23 |  | 26 | 23 |  | 26 | 32 |  | 20 | 18 |  | 16 | 16 |  | 24 | 14 |  | 20 | 29 |  | 15 | 19 |  | 20 | 31 | 392 |
| Married | 44 | 42 |  | 37 | 31 |  | 34 | 40 |  | 35 | 44 |  | 40 | 46 |  | 34 | 38 |  | 32 | 26 |  | 44 | 47 |  | 43 | 24 | 681 |
| Separated/divorced/widowed | 4 | 2 |  | 1 | 2 |  | 2 | 3 |  | 3 | 4 |  | 4 | 2 |  | 3 | 2 |  | 3 | 3 |  | 2 | 0 |  | 0 | 2 | 42 |
| **Have one or more children under 7 years old** | | |  |  |  |  |  |  |  |  |  |  |  |  |  |  |  |  |  |  |  |  |  |  |  |  | 0 |
| Yes | 17 | 30 |  | 20 | 20 |  | 19 | 20 |  | 17 | 22 |  | 14 | 27 |  | 17 | 17 |  | 14 | 18 |  | 24 | 28 |  | 26 | 12 | 362 |
| No | 51 | 37 |  | 44 | 36 |  | 43 | 55 |  | 41 | 44 |  | 46 | 37 |  | 44 | 37 |  | 41 | 40 |  | 37 | 38 |  | 37 | 45 | 753 |
