# Supplementary material for: Vaccine confidence in China after the Changsheng vaccine incident: a cross-sectional study
Source: BMC Public Health. 2019 Nov 27;19:1564. doi: 10.1186/s12889-019-7945-0 (PMC6880575; doi:10.1186/s12889-019-7945-0)
Supplement: Supplementary file 2 — Additional file 2: Table S1. The first cognitive factor "The consequences of the failure of the DTP Vaccination". Table S2. The second cognitive factor "The consequences of the failure of rabies vaccination". Table S3. The third cognitive factor "Satisfied with the response to this vaccine event". Table S4' The fourth cognitive factor "Concern about vaccine efficacy". Table S5. The fifth cognitive factor "Concern about vaccine safety". Table S6. The sixth cognitive factor “Vaccine is the most effective way of fighting infectious diseases”. Table S7. The seventh cognitive factor "Collapse of immunization defence can lead to outbreaks of infection diseases". Table S8. The eighth cognitive factor "Identifying the root of incident and strengthening regulations are important than punishments". Table S9. The ninth cognitive factor "Vaccination benefits outweigh risks". [file 12889_2019_7945_MOESM2_ESM.doc]

**Table 1** The first cognitive factor "The consequences of the failure of the DTP Vaccination".

|  | The consequences of the failure of the DTP Vaccination | | OR | p |
| --- | --- | --- | --- | --- |
|  | Not serious | Serious |  |  |
| Family income per month |  |  |  |  |
| ≤7000 Yuan | 38(16.24) | 84(9.53) |  |  |
| 7001-10000 Yuan | 46(19.66) | 202(22.93) | 1.987(1.206-3.273) | 0.007^**^ |
| 10001-13000 Yuan | 67(28.63) | 219(24.86) | 1.479(0.923-2.368) | 0.103 |
| 13001-16000 Yuan | 36(15.38) | 191(21.68) | 2.400(1.422-4.050) | 0.001^**^ |
| >16000 Yuan | 47(20.09) | 185(21.00) | 1.781(1.081-2.934) | 0.024^*^ |
| Education background |  |  |  |  |
| High school graduate or lower | 74(31.62) | 201(22.81) |  |  |
| Junior college | 35(14.96) | 142(16.12) | 1.494(0.947-2.356) | 0.085 |
| College graduate | 93(39.74) | 347(39.39) | 1.374(0.967-1.952) | 0.076 |
| Master and above | 32(13.68) | 191(21.68) | 2.197(1.388-3.479) | 0.001^**^ |

**Table 2** The second cognitive factor "The consequences of the failure of rabies vaccination".

|  | The consequences of the failure of rabies vaccination | | OR | p |
| --- | --- | --- | --- | --- |
|  | Not serious | Serious |  |  |
| Family income per month |  |  |  |  |
| ≤7000 Yuan | 29(21.80) | 93(9.47) |  |  |
| 7001-10000 Yuan | 23(17.29) | 225(22.91) | 3.050(1.677-5.548) | 0.000^**^ |
| 10001-13000 Yuan | 32(24.06) | 254(25.87) | 2.475(1.420-4.315) | 0.001^**^ |
| 13001-16000 Yuan | 30(22.56) | 197(20.06) | 2.048(1.162-3.609) | 0.013^*^ |
| >16000 Yuan | 19(14.29) | 213(21.69) | 3.496(1.866-6.548) | 0.000^**^ |
| Education background |  |  |  |  |
| High school graduate or lower | 45(33.83) | 230(23.42) |  |  |
| Junior college | 21(15.79) | 156(15.89) | 1.453(0.833-2.535) | 0.188 |
| College graduate | 50(37.59) | 390(39.71) | 1.526(0.988-2.356) | 0.057 |
| Master and above | 17(12.78) | 206(20.98) | 2.371(1.316-4.272) | 0.004^**^ |

**Table 3** The third cognitive factor "Satisfied with the response to this vaccine event".

|  | Satisfied with the response to this vaccine event | | OR | p |
| --- | --- | --- | --- | --- |
|  | Not satisfied | Satisfied |  |  |
| Family income per month |  |  |  |  |
| ≤7000 Yuan | 68(11.18) | 54(10.65) |  |  |
| 7001-10000 Yuan | 139(22.86) | 109(21.50) | 0.987(0.638-1.528) | 0.955 |
| 10001-13000 Yuan | 155(25.49) | 131(25.84) | 1.064(0.695-1.630) | 0.775 |
| 13001-16000 Yuan | 124(20.39) | 103(20.32) | 1.046(0.672-1.628) | 0.842 |
| >16000 Yuan | 122(20.07) | 110(21.70) | 1.135(0.731-1.764) | 0.572 |
| Education background |  |  |  |  |
| High school graduate or lower | 141(23.19) | 134(26.43) |  |  |
| Junior college | 102(16.78) | 75(14.79) | 0.774(0.529-1.132) | 0.186 |
| College graduate | 256(42.11) | 184(36.29) | 0.756(0.559-1.024) | 0.071 |
| Master and above | 109(17.93) | 114(22.49) | 1.101(0.773-1.567) | 0.595 |

**Table 4** The fourth cognitive factor "Concern about vaccine efficacy".

|  | Concern about vaccine efficacy | | | OR | p |
| --- | --- | --- | --- | --- | --- |
|  | Not worried | Worried | |  |  |
| Family income per month |  | |  |  |  |
| ≤7000 Yuan | 53(14.13) | | 69(9.32) |  |  |
| 7001-10000 Yuan | 84(22.40) | | 164(22.16) | 1.500(0.962-2.338) | 0.074 |
| 10001-13000 Yuan | 100(26.67) | | 186(25.14) | 1.429(0.927-2.202) | 0.106 |
| 13001-16000 Yuan | 64(17.07) | | 163(22.03) | 1.956(1.235-3.099) | 0.004^**^ |
| >16000 Yuan | 74(19.73) | | 158(21.35) | 1.640(1.044-2.577) | 0.032^*^ |
| Education background |  | |  |  |  |
| High school graduate or lower | 100(26.67) | | 175(23.65) |  |  |
| Junior college | 53(14.13) | | 124(16.76) | 1.337(0.892-2.004) | 0.160 |
| College graduate | 145(38.67) | | 295(39.86) | 1.163(0.848-1.595) | 0.350 |
| Master and above | 77(20.53) | | 146(19.73) | 1.083(0.749-1.568) | 0.671 |

**Table 5** The fifth cognitive factor "Concern about vaccine safety".

|  | Concern about vaccine safety | | OR | p |
| --- | --- | --- | --- | --- |
|  | Not worried | Worried |  |  |
| Family income per month |  |  |  |  |
| ≤7000 Yuan | 50(12.89) | 72(9.90) |  |  |
| 7001-10000 Yuan | 100(25.77) | 148(20.36) | 1.028(0.661-1.598) | 0.903 |
| 10001-13000 Yuan | 108(27.84) | 178(24.48) | 1.145(0.742-1.764) | 0.541 |
| 13001-16000 Yuan | 60(15.46) | 167(22.97) | 1.933(1.213-3.080) | 0.006^**^ |
| >16000 Yuan | 70(18.04) | 162(22.28) | 1.607(1.018-2.538) | 0.042^*^ |
| Education background |  |  |  |  |
| High school graduate or lower | 103(26.55) | 172(23.66) |  |  |
| Junior college | 49(12.63) | 128(17.61) | 1.564(1.038-2.357) | 0.032^*^ |
| College graduate | 151(38.92) | 289(39.75) | 1.146(0.838-1.568) | 0.394 |
| Master and above | 85(21.91) | 138(18.98) | 0.972(0.675-1.399) | 0.880 |

**Table 6** The sixth cognitive factor “Vaccine is the most effective way of fighting infectious diseases”.

|  | Vaccine is the most effective way of fighting infectious diseases | | OR | p |
| --- | --- | --- | --- | --- |
|  | Disagree | Agree |  |  |
| Family income per month |  |  |  |  |
| ≤7000 Yuan | 65(14.98) | 57(8.37) |  |  |
| 7001-10000 Yuan | 108(24.88) | 140(20.56) | 1.478(0.957-2.285) | 0.078 |
| 10001-13000 Yuan | 113(26.04) | 173(25.40) | 1.746(1.139-2.677) | 0.011^*^ |
| 13001-16000 Yuan | 82(18.89) | 145(21.29) | 2.016(1.290-3.153) | 0.002^**^ |
| >16000 Yuan | 66(15.21) | 166(24.38) | 2.868(1.818-4.525) | 0.000^***^ |
| Education background |  |  |  |  |
| High school graduate or lower | 112(25.81) | 163(23.94) |  |  |
| Junior college | 76(17.51) | 101(14.83) | 0.913(0.623-1.339) | 0.642 |
| College graduate | 173(39.86) | 267(39.21) | 1.060(0.780-1.442) | 0.708 |
| Master and above | 73(16.82) | 150(22.03) | 1.412(0.976-2.042) | 0.067 |

**Table 7** The seventh cognitive factor "Collapse of immunization defence can lead to outbreaks of infection diseases"

|  | Collapse of immunization defence can lead to outbreaks of infection diseases | | OR | p |
| --- | --- | --- | --- | --- |
|  | Disagree | Agree |  |  |
| Family income per month |  |  |  |  |
| ≤7000 Yuan | 55(15.71) | 67(8.76) |  |  |
| 7001-10000 Yuan | 96(27.43) | 152(19.87) | 1.300(0.838-2.016) | 0.241 |
| 10001-13000 Yuan | 81(23.14) | 205(26.80) | 2.078(1.338-3.225) | 0.001^**^ |
| 13001-16000 Yuan | 67(19.14) | 160(20.92) | 1.960(1.242-3.095) | 0.004^**^ |
| >16000 Yuan | 51(14.57) | 181(23.66) | 2.913(1.815-4.675) | 0.000^***^ |
| Education background |  |  |  |  |
| High school graduate or lower | 110(31.43) | 165(21.57) |  |  |
| Junior college | 54(15.43) | 123(16.08) | 1.519(1.017-2.267) | 0.041^*^ |
| College graduate | 137(39.14) | 303(39.61) | 1.474(1.077-2.019) | 0.016^*^ |
| Master and above | 49(14.00) | 174(22.75) | 2.367(1.590-3.526) | 0.000^**^ |

**Table 8** The eighth cognitive factor "Identifying the root of incident and strengthening regulations are important than punishments".

|  | Identifying the root of an incident and strengthening regulations are more important than punishments | | | OR | p |
| --- | --- | --- | --- | --- | --- |
|  | Disagree | Agree | |  |  |
| Family income per month |  | |  |  |  |
| ≤7000 Yuan | 42(12.14) | | 80(10.40) |  |  |
| 7001-10000 Yuan | 90(26.01) | | 158(20.55) | 0.922(0.585-1.452) | 0.725 |
| 10001-13000 Yuan | 90(26.01) | | 196(25.49) | 1.143(0.730-1.792) | 0.559 |
| 13001-16000 Yuan | 63(18.21) | | 164(21.33) | 1.367(0.851-2.194) | 0.196 |
| >16000 Yuan | 61(17.63) | | 171(22.24) | 1.472(0.916-2.365) | 0.110 |
| Education background |  | |  |  |  |
| High school graduate or lower | 96(27.75) | | 179(23.28) |  |  |
| Junior college | 64(18.50) | | 113(14.69) | 0.947(0.638-1.405) | 0.786 |
| College graduate | 129(37.28) | | 311(40.44) | 1.293(0.937-1.784) | 0.118 |
| Master and above | 57(16.47) | | 166(21.59) | 1.562(1.058-2.307) | 0.025^*^ |

**Table 9** The ninth cognitive factor "Vaccination benefits outweigh risks".

|  | Vaccination benefits outweigh risks | | OR | p |
| --- | --- | --- | --- | --- |
|  | Disagree | Agree |  |  |
| Family income per month |  |  |  |  |
| ≤7000 Yuan | 74(12.67) | 48(9.04) |  |  |
| 7001-10000 Yuan | 141(24.14) | 107(20.15) | 1.170(0.752-1.820) | 0.486 |
| 10001-13000 Yuan | 155(26.54) | 131(24.67) | 1.303(0.846-2.006) | 0.229 |
| 13001-16000 Yuan | 121(20.72) | 106(19.96) | 1.351(0.864-2.112) | 0.188 |
| >16000 Yuan | 93(15.92) | 139(26.18) | 2.304(1.472-3.607) | 0.000^**^ |
| Education background |  |  |  |  |
| High school graduate or lower | 183(31.34) | 92(17.33) |  |  |
| Junior college | 107(18.32) | 70(13.18) | 1.301(0.879-1.926) | 0.188 |
| College graduate | 217(37.16) | 223(42.00) | 2.044(1.495-2.794) | 0.000^**^ |
| Master and above | 77(13.18) | 146(27.50) | 3.772(2.598-5.475) | 0.000^**^ |
